# Comprehensive Study of Habitat Substrate-Related Variability of *Cotinus coggygria* Scop. as a Valuable Source of Natural Bioactive Compounds

**DOI:** 10.3390/plants14172695

**Published:** 2025-08-28

**Authors:** Milan Stanković, Nenad Zlatić, Marcello Locatelli, Miryam Perrucci, Tatjana Marković, Dragana Jakovljević

**Affiliations:** 1 Department of Biology and Ecology, Faculty of Science, University of Kragujevac, Radoja Domanovića 12, 34000 Kragujvac, Serbia; mstankovic@kg.ac.rs (M.S.); dragana.jakovljevic@pmf.kg.ac.rs (D.J.); 2 Department of Science, University “G. d’Annunzio” of Chieti-Pescara, 66100 Chieti, Italy; marcello.locatelli@unich.it; 3 Department of Biosciences and Agro-Food and Environmental Technologies, University of Teramo, 64100 Teramo, Italy; mperrucci@unite.it; 4 Department of Innovative Technologies in Medicine & Dentistry, University “G. d’Annunzio” of Chieti-Pescara, 66100 Chieti, Italy; 5 Institute for Medicinal Plant Research “Dr Josif Pančić”, Tadeuša Košćuška 1, 11000 Belgrade, Serbia; tmarkovic@mocbilja.rs

**Keywords:** smoked tree, bioactive compounds, inter-population variability, essential oils

## Abstract

*Cotinus coggygria* is a widespread medicinal and aromatic species known for its ecological plasticity, pharmacological potential, and cultivation prospects. Despite its broad distribution across heterogeneous habitats, little is known about how local ecological and pedochemical factors influence its physiological traits and secondary metabolite production. This study addresses this knowledge gap by analyzing the eco-physiological and phytochemical variability of *C. coggygria* across six natural populations differing in substrate type and geochemical conditions. The research reveals significant inter-population variability in element accumulation, oxidative stress markers, morphometric traits, and the qualitative and quantitative composition of essential oils and phenolic compounds. Soil analyses demonstrated notable differences in element concentrations (e.g., Ca, Fe, Co, Zn) across localities, correlating with geochemical conditions. Morphological traits, such as leaf size and petiole length, varied significantly, with pronounced differences observed in plants from thermophilous and metalliferous habitats. Oxidative stress, indicated by malondialdehyde (MDA) levels, was highest in populations from thermophilous habitats. Phenolic compound analysis revealed locality-specific differences, with plants from thermophilous habitats exhibiting the highest concentrations of gallic acid, catechin, and rutin. Essential oil yield and composition also varied: leaves from metalliferous habitats had the highest monoterpene hydrocarbon content, while bark samples from thermophilous habitats showed elevated sesquiterpene levels. This comprehensive analysis underscores the interplay between habitat-specific conditions and the physiological and biochemical processes of *C. coggygria*. The findings provide valuable insights for optimizing substrate conditions and ecological management, with implications for the cultivation of the species to enhance the synthesis of bioactive compounds. These results support sustainable land use practices and the development of high-value plant-based products, offering significant implications for agriculture, pharmacology, and ecosystem restoration. Future studies should further explore the genetic and biochemical mechanisms underlying this species’ adaptability and resource optimization in heterogeneous environments.

## 1. Introduction

In their natural habitats, plants are exposed to a complex of abiotic and biotic factors. Their influence varies spatially and temporally, with a combined effect on plants. During evolution, plant species adapt their life form to habitat conditions by adjusting physiological properties like primary and secondary metabolism. Products of plant secondary metabolism have significant value and are used in various purposes. Medicinal aromatic plants and natural products have been used for centuries worldwide as traditional treatments for various health ailments. In rural areas of developing countries, these traditional methods continue to serve as the primary source of medical care as a suitable and accessible alternative, especially in regions with limited access to modern medical facilities and pharmaceuticals. The use of medicinal plants in traditional medicine is deeply rooted in the cultural heritage and everyday practices of many communities. These natural remedies often consist of complex mixtures of bioactive compounds that work synergistically to provide effective and safe therapeutic effects. Unlike many modern pharmaceuticals, these bioactive compounds can target multiple biological processes. The renewed interest in natural and sustainable sources of treatment has led to increased research in the field of phytomedicine [1,2,3,4].

*Cotinus coggygria* Scop. (syn. *Rhus cotinus* L., fam. Anacardiaceae) is a deciduous flowering plant, the multi-branched above-ground part of which varies from a low shrub with prostrate or erect branches to a small tree (2–5 m). The leaves (5–10 cm in diameter) are broadly elliptic to obovate with a waxy glaucous surface. Autumn leaf coloration varies from yellow to purple-red. In the flowering and fruiting period, this plant acquires a specific habitus. *C. coggygria* is widespread in temperate biomes, as well as the Mediterranean [5]. The area of native distribution covers territory from southern Europe to southern and central China [6]. The most common habitats of this species are canyons and gorges, as well as sandy deposits with variable pedochemical compositions and water regimes. *C. coggygria* (also known as the “smoke tree”) is frequently used in horticulture for its attractive inflorescences, infructescences, and leaf color. *Cotinus coggygria* Scop. has a long history of use in traditional medicine across Europe and Asia, and its medicinal properties have been the subject of growing pharmacological interest in recent decades [7,8,9,10,11]. Traditionally, various parts of the plant have been used in the form of infusions, decoctions, and topical applications for the treatment of inflammation, infections, skin conditions, and gastrointestinal disorders [10,11]. In Serbian ethnomedicine, bark decoctions have also been employed in the treatment of cancer, prompting contemporary interest in its potential anticancer effects [11]. Phytochemical studies have revealed that *C. coggygria* is rich in bioactive compounds, including flavonoids (quercetin, myricetin, fisetin), phenolic acids (gallic, chlorogenic, and ellagic acid), tannins, stilbenes, and essential oils [12,13]. Fisetin, a major flavonol in this species, has demonstrated potent antioxidant, anti-inflammatory, and antiproliferative properties [13]. The essential oil, particularly from the leaves and young shoots, contains high levels of monoterpenes and sesquiterpenes such as α-pinene, β-caryophyllene, and germacrene D, compounds widely recognized for their antimicrobial and anti-inflammatory activity [13]. Numerous in vitro and in vivo studies have confirmed that extracts of *C. coggygria* exhibit significant biological activities, including antioxidant, anti-inflammatory, antimicrobial, hepatoprotective, and cytotoxic effects against various cancer cell lines [11,12,13].

Plant growth and development depends on various inorganic elements. Different forms and amounts of inorganic elements can be found both in soil and in plants, which can affect plants’ main components and take part in different physiological processes [14,15]. Based on specific distribution and habitat preferences that include different types of substrate, especially in terms of pedochemical composition, *C. coggygria* is a suitable medicinal plant for the intra-specific analysis of ecophysiological traits among different populations from various habitat conditions. Additionally, it is known that the inorganic elements in soil and overall habitat conditions have a significant impact on the qualitative properties of plants, which can be determined by the composition and content of elements, as well as by the synthesis and alterations of bioactive compounds. In this regard, we conducted a comparative analysis of the selected habitat characteristics and ecophysiological traits, such as the substrate and plant material metal content and stress parameters, in addition to quantitative and qualitative analyses of essential oils and phenolic compounds in leaves and bark as well as morphometric characteristics for the species *C. coggygria* sampled from six different natural habitats. This will provide basic theoretical data on microclimatic habitat conditions, substrate characteristics for planting demands, and correlations of these characteristics with the synthesis and activity of main bioactive compounds.

## 2. Results and Discussion

### 2.1. Content of Major and Trace Elements

#### 2.1.1. The Quantity of Elements in the Soil Samples

The results of the analysis of the quantity of elements Al, As, B, Ba, Ca, Cd, Co, Cr, Cu, Fe, K, Li, Mg, Mn, Na, Ni, P, Pb, S, Se, and Zn in the soil samples from different localities are presented in Table 1.

The mean values were ordered in the following way: Ca > Fe > Al > Mg > K > P > Mn > S > Ni > Cr > Li > Zn > Na > Ba > Co > Cu > Pb > As > B > Cd > Se. Soil samples from the BG locality were characterized by the highest values of Co, Fe, and Zn. Soil samples from the IG locality were characterized by the highest values of B, Cd, Cr, Cu, Mg, Mn, Na, Ni, P, Pb, and S. Soil samples from the OKG locality were characterized by the highest values of Ba. Soil samples from the GRG locality were characterized by the highest values of Al and K, while soil samples from the DG locality were characterized by the highest values of As, Ca, and Li. The element Se was not detected in the soil samples.

#### 2.1.2. The Quantity of Elements in Leaves and Bark Samples

The results of the analysis of the quantity of elements Al, As, B, Ba, Ca, Cd, Co, Cr, Cu, Fe, K, Li, Mg, Mn, Na, Ni, P, Pb, S, Se, and Zn in the leaves of *C. coggygria* are presented in Table 1.

The mean values were ordered as follows: Ca > K > P > S > Mg > Fe > B > Zn > Mn > Na > Ba > Al > Li > Cu > Ni > Cr > Se > Pb > Cd > As > Co. Leaf samples of *C. coggygria* from the IG locality are characterized by the highest values of Al, Cd, Cr, Fe, Mg, and Ni. Leaf samples of examined species from the OKG locality are characterized by the highest values of Ba and S, while leaf samples from the GRG locality are characterized by the highest values of Cu, Mn, Na, Pb, and Zn. Leaf samples of *C. coggygria* from DG locality are characterized by the highest values of B, Ca, K, Li, and P, while samples from DSD locality are characterized by the highest values of Se. Elements such as As and Co were not detected.

The results of the analysis of the quantity of selected elements in the bark samples of *C. coggygria* are presented in Table 1.

The order of elements in the bark was as follows: Ca > K > P > Mg > S > Na > Fe > Ba > Zn > Al > B > Mn > Li > Cu > Ni > Cr > Se > Pb > Co > Cd > As. The *C. coggygria* samples of bark from the BG locality are characterized by the highest values of Co, Ni, and S, while bark samples from the IG locality had the highest values of Fe, Mg, and Zn. Bark samples from the OKG locality had the highest values of Ba and Ca, while bark samples from the GRG locality had the highest values of Al, Cd, Cu, Na, and Pb. Bark samples of *C. coggygria* from the DG locality are characterized by the highest values of Cr, K, Li, P, and Se, while bark samples of *C. coggygria* from the DSD locality are characterized by the highest values of B and Mn. The element As was not detected.

It is known that elements like Ca, K, S, Mg, and P are most abundant in plant material, while elements with essential functionality in micro content like Cr, Li, Cu, Fe, Mn, and Zn are less abundant [16]. Plants can accumulate macro biogenic elements like K, Ca, Mg, S, and P to suppress the influence of toxic metals and to secure stabilized physiological function in specific habitats. The presence of certain elements in the soil is determined by the composition of elements in the parent material, as well as by the effect of biological and climatic factors. The diversity of these factors influences the differences in the presence and quantity of certain elements in the same and different types of soil [16]. In the context of botanical and ecological research, indicator elements represent important data for precise assessment and analysis of the biopotential of different plant species in various habitats. Based on the results presented here, it can be noticed that the content of elements in the soil is in relation with elements in bark and leaves of *C. coggygria*. The leaf samples from BG and IG localities are distinct from other samples due to the higher content of Ni in leaves and Ni and Co in the bark. In leaf samples, As and Co were not detected in any of the samples from different localities. Similarly, As was not detected in bark samples from any locality, whereas Co was detected in the bark samples from BG, IG and DG localities. Notably, the contents of Ca and Li were significantly lower in both leaf and bark samples from BG and IG compared to other localities. This discrepancy suggests a unique geochemical influence in these localities, potentially related to the specific soil composition and underlying geological formations, which may affect the uptake and accumulation of these elements in the plant tissues [16].

### 2.2. Variability of Leaf Morphology

The variation in leaf blade length among specimens of the *C. coggygria* collected from different localities ranges from 64.37 to 76.71 mm (Table 2). Similarly, the variability in leaf blade width ranges from 45.18 to 57.76 mm. Leaf area measurements exhibit a range of 2075.99 to 3550.65 mm^2^. Additionally, the variability in leaf petiole length ranges from 15.95 to 32.47 mm within the species examined. The leaves of *C. coggygria* collected from the GRG locality exhibit distinctive features in terms of their length, width, and overall leaf area, whereas leaves of *C. coggygria* from the BG locality possess the longest leaf petioles. Numerous studies have demonstrated that differences in leaf morphology result from adaptations to specific growth environments [17,18]. The morphological differentiation observed in the leaf traits of *Cotinus coggygria* across various localities can be attributed, at least in part, to differences in climatic conditions, particularly temperature and precipitation parameters. Notably, specimens from the GRG locality exhibited the most developed leaf blades in terms of length, width, and total area. According to the climatic data, GRG is among the cooler localities, with a relatively low annual mean temperature (bio1 = 9.8 °C) and the lowest mean temperature of the coldest quarter (bio11 = 0.2 °C). Despite this, it also has moderately high temperature seasonality (bio4 = 758.3) and relatively low precipitation, particularly in the driest quarter (bio17 = 136 mm) and the coldest quarter (bio19 = 147 mm).

This combination of cooler temperatures and moderate moisture availability may promote greater leaf surface expansion in order to optimize photosynthetic efficiency under potentially limiting light or temperature conditions. Furthermore, although GRG has the lowest annual precipitation among the high-altitude localities (bio12 = 693 mm), it receives considerable precipitation during the warmest quarter (bio18 = 205 mm), which may coincide with the peak vegetative growth period, supporting the development of larger leaves. In contrast, the BG population, which exhibited the longest petioles, is characterized by slightly higher annual temperatures (bio1 = 10.6 °C) and moderate annual precipitation (bio12 = 793 mm). However, BG also has the highest precipitation during the coldest quarter (bio19 = 172 mm) and a relatively balanced distribution of rainfall throughout the year (lower precipitation seasonality; bio15 = 19.7). The elongated petioles in this population might reflect an adaptive trait allowing more efficient leaf positioning in relatively denser vegetation or microclimatic conditions that favor increased light interception. The DSD locality, which likely corresponds to specimens with smaller leaf blades, represents the warmest (bio1 = 11.5 °C; bio10 = 20.6 °C) and driest site (lowest annual precipitation, bio12 = 639 mm; lowest values across bio16–bio19). Here, the lower water availability and elevated temperatures during the growing season (e.g., bio18 = 201 mm) may constrain leaf size development. Smaller leaves in such conditions help reduce transpirational water loss and are commonly considered an adaptive response to xeric environments. Interestingly, the OKG population, situated in one of the coolest and wettest areas (bio1 = 9.8 °C; bio12 = 851 mm; bio18 = 242 mm), might also be expected to develop larger leaf structures. However, depending on the observed morphology in this group (not specified here), it could serve as a reference to assess whether precipitation alone, or in combination with temperature, better explains the leaf size variation. If the OKG leaves are not as large as those from GRG despite higher moisture availability, then temperature might be the more limiting factor for growth. The spatial variability in *C. coggygria* leaf traits is likely influenced by a complex interplay of climatic factors. Larger leaf blades are associated with cooler and moderately moist environments (GRG locality), whereas longer petioles may reflect microhabitat-driven plasticity (BG locality), and smaller leaves appear in warmer, drier settings (DSD locality).

### 2.3. Lipid Peroxidation

**Figure 1:**
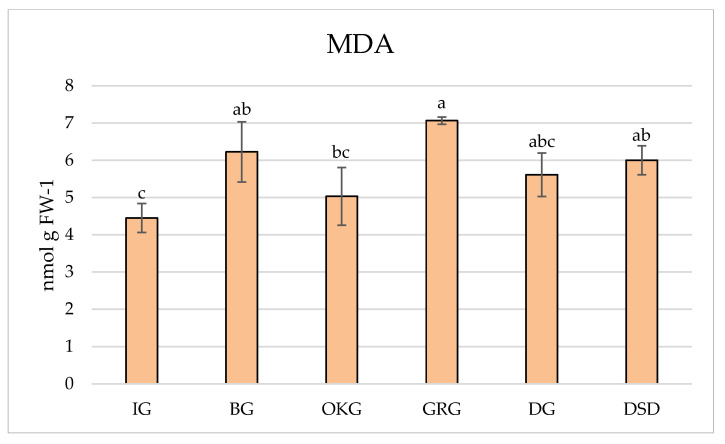
MDA content in the leaves of *C. coggygria* from different locations. Serpentinite habitat (BG, IG); limestone habitat (OKG, GRG, DG); sandy habitat (DSD). Bar represents mean value ± standard deviation (SD). Means sharing the same letter do not differ significantly by Tukey’s test (*p* ≥ 0.05).

In studies related to oxidative stress and plant abiotic and biotic stress responses, the measurement of MDA is used as a lipid peroxidation marker, where the quantification of lipid peroxidation is estimated by measuring the concentration of MDA as a secondary oxidation product [19,20]. In the present study, the level of lipid peroxidation varied in leaves of *C. coggygria* collected from different localities (Figure 1). The highest level of MDA was measured in leaves from plants grown in GRG, followed by BG locality, whereas the lowest level of MDA was detected for plants collected from IG locality. The MDA production is used as a general indicator of the degree of oxidative stress-induced lipid peroxidation. Plants experiencing oxidative stress increase lipid peroxidative products as a result of the cytotoxic product of lipid peroxidation (MDA), which is produced when excessive free radical formation occurs. Further deterioration of biological membranes’ structural and functional integrity, increased plasma membrane permeability, K+ ion leakage, amino acid oxidation, and ultimately cell death can be caused by lipid peroxidation [21]. Based on the obtained results, GRG and BG localities can be regarded as less favorable, while IG is the most favorable locality for *C. coggygria* growth and development.

### 2.4. Activity of Phenylalanine Ammonia-Lyase (PAL)

**Figure 2:**
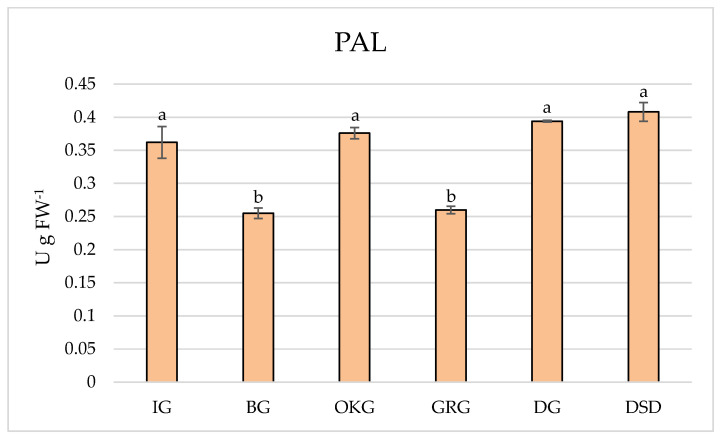
PAL activity in the leaves of *C. coggygria* from different locations. Serpentinite habitat (BG, IG); limestone habitat (OKG, GRG, DG); sandy habitat (DSD). Bar represents mean value ± standard deviation (SD). Means sharing the same letter do not differ significantly by Tukey’s test (*p* ≥ 0.05).

Significant variation in PAL activity was recorded in leaves of *C. coggygria* collected from different localities (Figure 2). The lowest activities and the strongest location-dependent effects were estimated for plants grown in GRG locality, followed by plants grown in BG locality, whereas for other investigated localities no significant variation in PAL activity can be noticed. DG and DSD are localities where the highest activity of PAL was recorded in leaves of *C. coggygria*. The initial stage of the phenylpropanoid pathway, which generates precursors to numerous significant secondary metabolites, is catalyzed by PAL [22]. PAL catalyzes the conversion of phenylalanine to trans-cinnamic acid, which then directs metabolic flow from the shikimate pathway to the various branches of phenylpropanoid metabolism. A variety of factors influence how phenylpropanoid metabolism is regulated and how stress and subsequent developmental processes affect phenylpropanoid homeostasis in plants [23]. Recent research confirmed that the metabolism of phenols is metal-specific, as it is impacted by the physical variations between the metals, whereas various impacts of the metals on the formation and accumulation of phenolic compounds can be demonstrated, indicating that the phenylpropanoid pathway has a distinct role in the synthesis of secondary metabolites besides phenolic substances [24].

### 2.5. Content of Secondary Metabolites

#### 2.5.1. Determination of Individual Phenolic Compounds

Table 3 reports the phenolics quantitative data in mg g^−1^ (±SD) observed for the different plant extracts. In all extracts, gallic acid, catechin and rutin were detected.

Chlorogenic acid was detected in leaf samples collected from BG, IG, OKG, DG and DSD localities; however, in leaf samples collected from IG locality the content was below the Limit of Quantification (0.20 mg g^−1^). Epicatechin was presented in leaf samples collected from BG, OKG, GRG and DSD localities and bark samples collected from IG, OKG, DG and DSD. *o*-coumaric acid was detected and quantified only in the leaf extracts. In general, among the tested phenolic compounds, catechin was most abundant in both leaves and bark samples, with the highest values determined from leaf samples collected from DG and DSD localities. On the contrary, GRG and IG can be marked as the localities with the lowest *C. coggygria* quantity and quality among the tested phenolic compounds. Research on the influence of abiotic stress on catechin synthesis and accumulation is primarily focused on the effects of light and water regimes in habitats [25]. Exposure to higher light intensity leads to increased biosynthesis of catechin in *Camellia sinensis* [25]. On the other hand, catechin influences the growth of primary and lateral roots, increases leaf surface area, and contributes to the formation of a continuous xylem ring in *Camellia sinensis*, enhancing nutrient absorption [26]. This compound exhibits pronounced antioxidant activity, neutralizing reactive oxygen species and chelating heavy metal ions. The antioxidant activities of catechin include the inhibition of pro-oxidative enzymes and the induction of antioxidant enzymes [27]. Generally, the results obtained via phenolic screening can be of great importance in assessing the medicinal potential of the species *C. coggygria*, considering its wide application in traditional medicine. This plant species can be considered a potential source of certain phenolic compounds and can be utilized as a raw material for their extraction. Still, based on the results obtained, the collection of samples from different wild sources may limit further utilization.

**Figure 3:**
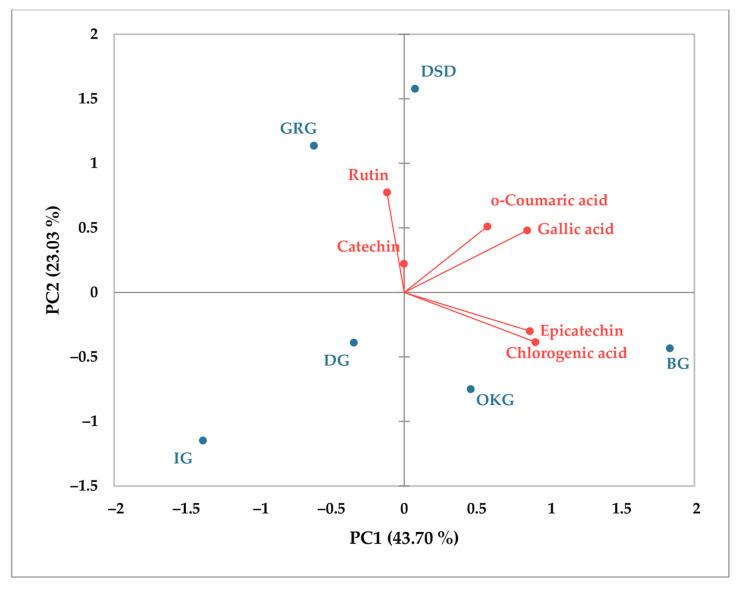
Principal component analysis (PCA) of main phenolic components in *C. coggygria* leaves.

**Figure 4:**
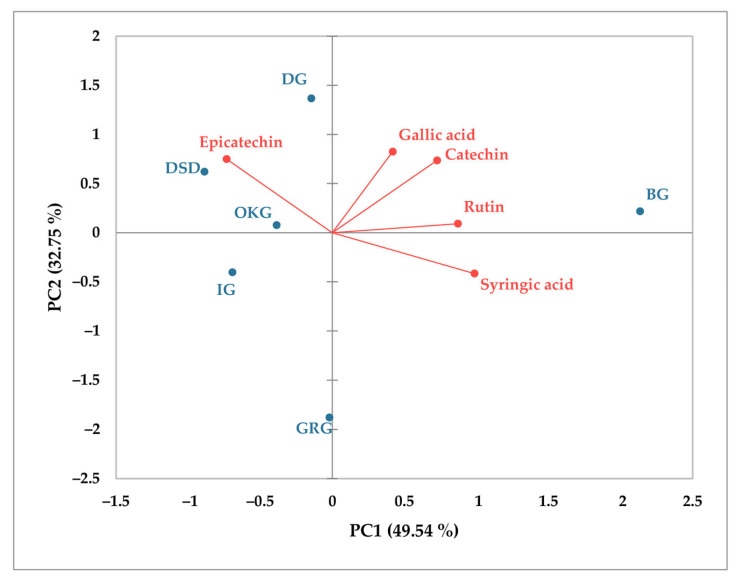
Principal component analysis (PCA) of main phenolic components in *C. coggygria* bark.

The amount of phenolic acids and flavonoids in leaves and bark of *C. coggygria* from different localities was further analyzed via principal component analysis (PCA) to provide the relationship between the content of these metabolites in various populations of *C. coggygria* and the investigated localities. Spatial representation for leaves (Figure 3) showed total variance accounting for 66.73%, elucidated by PC1 (43.70%) and PC2 (23.03%). Generally, there is a good relationship between gallic acid, *o*-coumaric acid, chlorogenic acid, and epicatechin, which is opposite to rutin and catechin. Spatial representation for bark (Figure 4) showed total variance accounting for 82.29%. A good relationship was determined between gallic acid, syringic acid, catechin, and rutin, while epicatechin was strongly negatively related to syringic acid. In both cases, major differences can be seen regarding the localities used in this study, with the most significant impact being that of the BG locality.

#### 2.5.2. Content of Total Phenolic Compounds and Flavonoids and Total Antioxidant Activity of *C. coggygria*

The content of total phenolic compounds in the *C. coggygria* leaf extracts ranged from 452.56 to 526.47 mg GAE g^−1^ of sample (Table 4). The leaf samples collected from the IG and DSD localities had the highest amount of total phenolic compounds. The quantity of flavonoids in the leaf extracts ranged from 54.99 to 72.43 mg RUE g^−1^ of sample. The leaf samples collected from the GRG locality had the highest amount of total flavonoids. The results of the total antioxidant activity of methanol leaf extracts ranged from 1219.76 to 210.56 μg mL^−1^, while the results of the antioxidant activity of leaf essential oil ranged from 3.89 to 0.70%. The extract of *C. coggygria* and the sample of essential oil from the GRG locality were distinguished for their strongest ability to neutralize free radicals compared to other samples (Table 4).

The amount of total phenolic compounds in the samples of *C. coggygria* bark extracts ranged from 186.32 to 218.99 mg GAE g^−1^ of sample (Table 4). The bark sample collected from the BG locality had the highest amount of total phenolic compounds. The quantity of flavonoids in the bark extracts of the investigated species ranged from 22.85 to 41.30 mg RUE g^−1^ of sample. The bark samples collected from the BG locality had the highest amount of total flavonoids. The results of the antioxidant activity of methanol bark extracts ranged from 143.07 to 31.48 μg mL^−1^, while the results of the antioxidant activity of bark essential oil ranged from 6.31 to 4.42%. The extract of *C. coggygria* from the DSD locality was distinguished for its strongest ability to neutralize free radicals, while the sample of essential oil of *C. coggygria* from the BG locality was distinguished for its strongest ability to neutralize free radicals compared to other samples (Table 4).

Scientific studies have shown that the presence of certain elements and their increased quantities in soil directly or indirectly influence the synthesis of secondary metabolites in plants [28]. Experimental evidence has demonstrated that certain plant species can increase the synthesis of phenolic compounds due to the higher presence of trace elements in the soil [29,30]. Phenolic compounds and other groups of secondary metabolites possess various pharmacological properties, so any change in the quantity or composition of phytochemical compounds can potentially affect the efficiency of plant products. Besides other mechanisms, the antioxidant capacity of secondary metabolites plays a significant role in the adaptation of plants to various abiotic factors, and it is known that imbalances in the water regime and temperature stress may lead to increased synthesis of plant secondary metabolites, including flavonoids [31,32]. Due to the soil structure and disturbed water regime, some soils are arid [33]. It is important to note that variability in the concentration of secondary metabolites and antioxidant activity can be attributed to specific habitat conditions, including different element quantities and thermal and water regimes of habitats [34]. These findings highlight the importance of habitat characteristics and abiotic factors in the quantitative composition of secondary metabolites and antioxidant properties of plants growing in different habitats [35].

### 2.6. Chemical Analysis of Essential Oils

The chemical composition of essential oils extracted from the leaves and bark of *C. coggygria* collected from six localities is presented in Table 5 and Table 6.

The average essential oil yield from *C. coggygria* leaf samples was 0.20%, with the highest yield of 0.25% recorded in samples from BG and IG localities. Monoterpene hydrocarbons (MH) were the dominant class of compounds in the leaf essential oils across all localities, ranging from 96.9% in BG to 92.6% in GRG. Oxygenated monoterpenes (OM) ranged from 1.4% in OKG to 0.9% in GRG. Sesquiterpene hydrocarbons (SH) varied from 5.9% in GRG to 1.4% in OKG, while oxygenated sesquiterpenes (OS) ranged from 0.3% in GRG to 0.1% in BG, OKG, and DSD. The predominant individual constituents in the leaf essential oils, based on average values, were limonene (52.44%), cis-*β*-ocimene (22.00%), terpinolene (5.27%), trans-*β*-ocimene (5.10%), myrcene (4.40%), *α*-pinene (4.26%), and trans-*β*-caryophyllene (1.46%) (Table 5).

The average essential oil yield from *C. coggygria* bark samples was higher than that of the leaves, at 0.72%, with the highest yield (1.00%) obtained from samples collected at OKG locality. Monoterpene hydrocarbons (MH) were the predominant class in bark essential oils, ranging from 93.4% in OKG to 81.02% in GRG. Oxygenated monoterpenes (OM) varied from 2.18% in BG to 0.68% in GRG, while sesquiterpene hydrocarbons (SH) ranged from 16.23% in GRG to 5.46% in OKG. Oxygenated sesquiterpenes (OS) were present from 2.44% in DG to 0.17% in OKG. The most abundant individual constituents in the bark essential oils, based on average values, were *α*-pinene (48.43%), *β*-pinene (14.25%), limonene (8.61%), germacrene D (7.58%), myrcene (3.98%), *β*-phellandrene (2.46%), α-thujene (2.20%), cis-*β*-ocimene (1.70%), and camphene (1.05%) (Table 6).

Previous studies have demonstrated considerable qualitative and quantitative variation in the composition of *C. coggygria* essential oils across different geographic regions. For example, essential oils from Turkish populations were dominated by limonene (48.5%), (*Z*)-*β*-ocimene (27.9%), and (*E*)-*β*-ocimene (9.7%) [8]. In contrast, Hungarian samples exhibited a broader profile, with limonene (30.0–40.0%), *α*-pinene (24.4–34.3%), *β*-pinene (7.6–20.2%), *δ*^3^-carene (4.6–11.0%), and *α*-terpinolene (3.3–10.6%) as major constituents [36]. Oils from Serbian populations were characterized by limonene (3.39–39.5%) and *α*-pinene (15.1–21.9%) [37], while those from Bulgaria contained predominantly α-pinene (44.0%), limonene (20.0%), and *β*-pinene (11.4%) [38]. Notably, essential oils from Greek populations showed substantial chemical diversity, with limonene (10.9–67.4%), *α*-pinene (14.7–15.9%), myrcene (14.0–32.0%), sabinene (18.0–24.2%), terpin-4-ol (10.9%), and terpinolene (8.6%) as the principal components [39]. Compared with previously reported chemotypes, our leaf oils most closely resemble the Turkish profile in limonene dominance (~52%), but differ by higher *cis*-*β*-ocimene (22%) and notable terpinolene (5.3%) and myrcene (4.4%) levels. In contrast to Hungarian populations rich in α-pinene (24–34%) and *β*-pinene (7–20%), our samples contained much lower proportions of these monoterpenes. Bark oils, dominated by α-pinene (48.4%) and *β*-pinene (14.3%), were similar to the Bulgarian chemotype but contained less limonene (8.6%) and more germacrene D (7.6%), indicating a distinct *α*-pinene–*β*-pinene–germacrene D type not previously documented.

Microclimatic factors presented in Table 7, including variations in temperature ranges, isothermality, precipitation seasonality, and temperature extremes, directly influence plant physiology, especially the production of secondary metabolites such as essential oils [40]. These variations underscore the significant influence of environmental conditions such as temperature, precipitation, and humidity on the biosynthesis of secondary metabolites [41]. For instance, higher mean temperatures during the driest and warmest quarters (DS locality) may induce oxidative stress, trigger antioxidant defense mechanisms, and stimulate biosynthetic pathways for volatile compounds as protective agents [42]. In contrast, cooler and more stable conditions (IG and OKG) may lead to lower stress levels and a different profile of essential oil constituents. Temperature seasonality and annual temperature range (bio4 and bio7), which are higher in GG and DG, can further contribute to metabolic fluctuations by affecting enzymatic activity and gene expression related to secondary metabolism. Precipitation patterns also play a critical role: localities with higher precipitation during the wettest quarters (OKG and IG) may support more vigorous vegetative growth and altered resource allocation, potentially diluting essential oil concentrations, while arid localities (DS and DG) may exhibit a concentration of certain bioactive compounds due to water-deficit-induced stress [43]. Such environmental conditions are known to modulate ROS (reactive oxygen species) production and antioxidant responses, thereby shaping both the composition and yield of essential oils. This is consistent with prior studies linking microclimatic stress conditions to enhanced synthesis of bioactive volatile compounds with antioxidant properties [41].

Edaphic factors (e.g., soil type, nutrient availability, pH) also affect root nutrient uptake and metabolic pathways, while orographic conditions (altitude, slope, and microclimate) contribute to habitat heterogeneity, potentially leading to distinct chemotypes even among geographically close populations. Identifying such chemically distinct ecotypes is valuable for selecting genotypes with specific bioactive properties that warrant further investigation for potential pharmaceutical or industrial applications, pending comprehensive in vivo validation and toxicity assessments.

**Figure 5:**
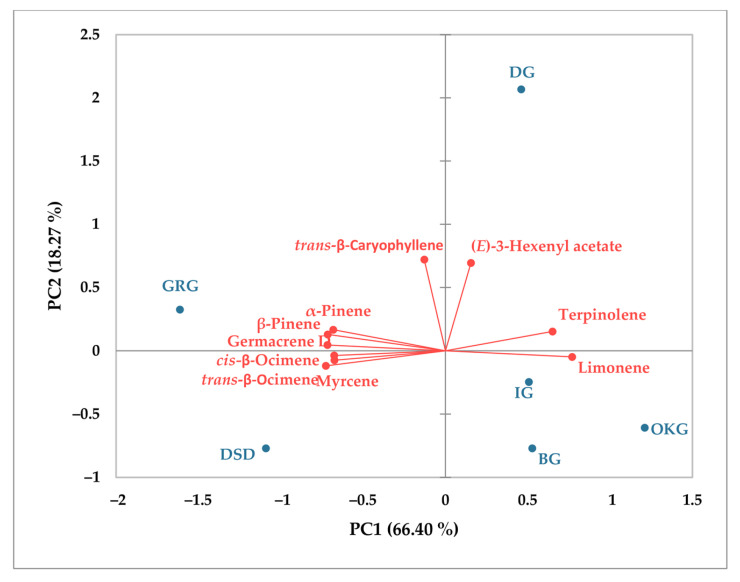
Principal component analysis (PCA) of main essential oil components in *C. coggygria* leaves.

**Figure 6:**
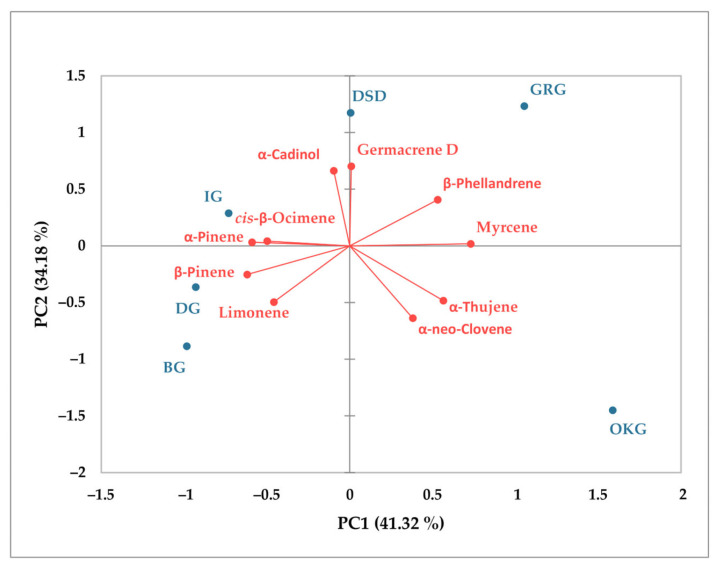
Principal component analysis (PCA) of main essential oil components in *C. coggygria* bark.

PCA was employed to assess the relationships between the ten main essential oil constituents in both leaves and bark across the different sampling sites. For leaf samples (Figure 5), the first two major components accounted for 84.67% of the total variance (66.40% and 18.27%, respectively). Samples from DG formed a distinct group with unique chemical characteristics. Leaf samples from IG, OKG, and BG clustered together on the positive side of PC1 and were closely associated with limonene content. In contrast, samples from GRG and DSD were strongly associated with *α*-pinene, *β*-pinene, myrcene, germacrene D, *cis*-*β*-ocimene, and *trans*-*β*-ocimene. In the case of bark samples (Figure 6), 75.50% of the observed variability was explained by the first two principal components, with PC1 accounting for 41.32% and PC2 for 34.18%. The OKG sample exhibited distinct chemical characteristics. A strong positive correlation was observed between *α*-pinene, *β*-pinene, *α*-thujene, and *α*-neo-cloven, while a strong negative correlation was found for limonene, *β*-phellandrene, *α*-neo-cloven, and germacrene D.

### 2.7. Correlation Analysis Between Inorganic Elements in Soil and Active Components in C. coggygria Leaves and Bark

**Figure 7:**
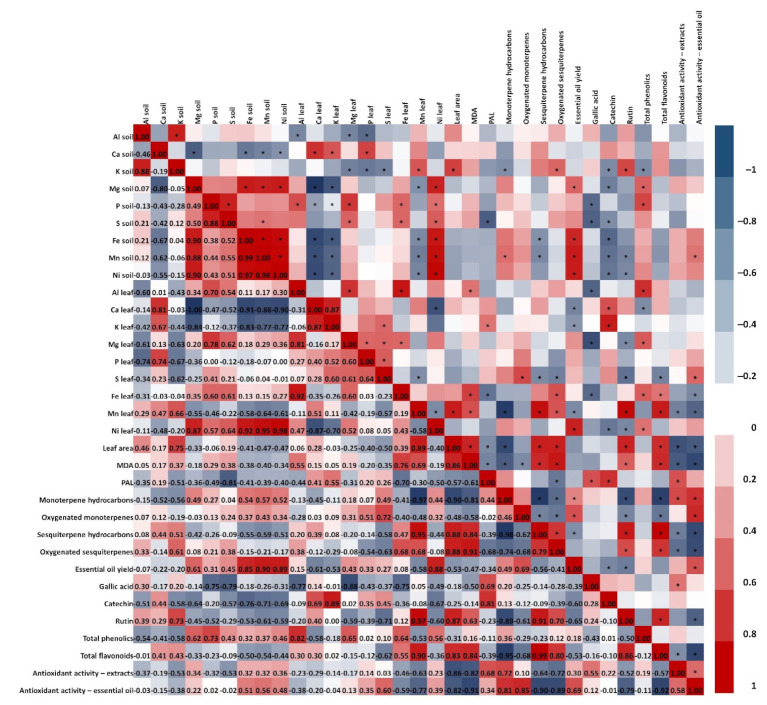
Correlation coefficient of main inorganic element content in soil, main inorganic elements in leaf of *C. coggygria*, leaf area, lipid peroxidation (MDA), PAL activity, and main active components in *C. coggygria* leaf. * *p* ≤ 0.05.

**Figure 8:**
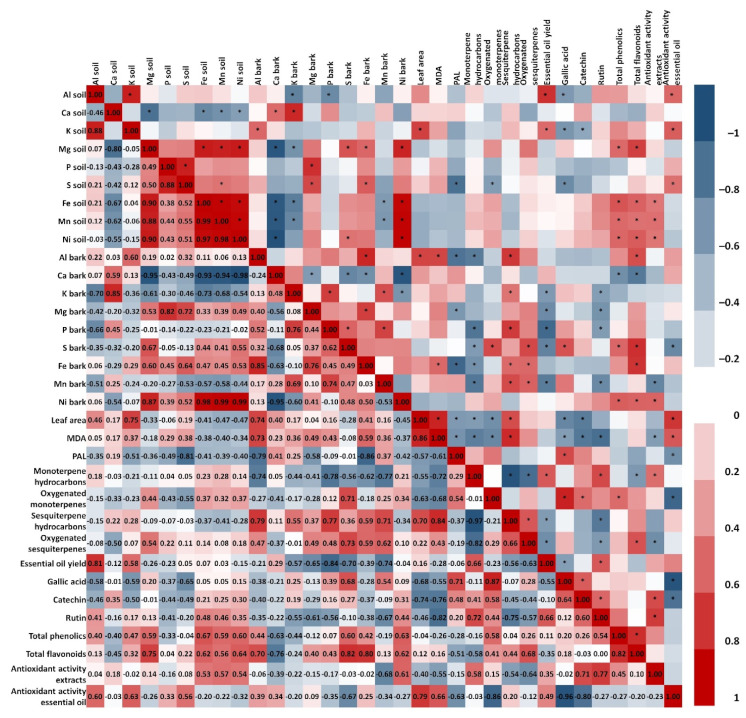
Correlation coefficient of main inorganic element content in soil, main inorganic elements in bark of *C. coggygria*, leaf area, lipid peroxidation (MDA), PAL activity, and main active components in *C. coggygria* bark. * *p* ≤ 0.05.

As shown in Figure 7 and Figure 8, the content of main inorganic elements in rhizosphere soil and in leaves and bark of *C. coggygria* showed a significant correlation. The content of Ca in soil was positively correlated with leaf Ca, K, Mg and P content and bark Ca and K content. The leaf Mg, P, and S content was negatively correlated with K in soil, while Mg content in soil was negatively correlated with both leaf and bark Ca and K content. Additionally, Ca and K content in leaves and bark showed a significant negative correlation with soil Fe, Mn, and Ni content. The positive correlation between soil Ca and leaves (Ca, K, Mg, P) as well as bark (Ca, K) is consistent with the role of Ca in improving nutrient uptake efficiency and maintaining membrane and cell wall stability in woody plants [44]. Negative correlations between soil K and leaf Mg, P, and S, as well as between soil Mg and leaf/bark Ca and K, reflect competitive uptake patterns well known in mineral nutrition [16]. High K can suppress Mg uptake, while elevated Mg or Ca can reduce K accumulation [45]. Furthermore, the observation that higher Ca and K in plant tissues coincided with lower Fe, Mn, and Ni in soil suggests antagonistic ion interactions, also described in medicinal shrubs [16].

Variability in leaf morphology, estimated through the measurement of leaf area, correlated positively with soil K content, Mn leaf content, and content of Al in bark. Additionally, higher values of leaf area pointed toward higher MDA content and lower PAL activity. Leaf area correlated positively with soil K, leaf Mn, and bark Al, and was associated with higher MDA and lower PAL activity, indicating oxidative stress-related morphological responses [46].

It can be seen that the content of inorganic elements in rhizosphere soil has a strong correlation with the accumulation of active components in both leaves and bark of *C. coggygria*. In leaves, essential oil yield correlated with soil Mg, Fe, Mn, and Ni content. Mg is an essential cofactor for terpene synthases and can enhance monoterpene biosynthesis [47]. Compound class-specific trends were evident: monoterpene hydrocarbons and oxygenated monoterpenes correlated negatively with leaf area and MDA, while sesquiterpene classes correlated positively, indicating possible biosynthetic shifts under stress [48]. Except for soil Al and K content, bark essential oil yield did not correlate with content of the rhizosphere inorganic elements. However, negative correlation can be seen among bark essential oil yield and bark K, Mg, P, Mn, and S content. Still, for these elements positive correlation was shown with particular classes of compounds—sesquiterpene hydrocarbons and oxygenated sesquiterpenes—while negative correlation was observed among monoterpene hydrocarbons and bark S, Fe, and Mn content. These nutrients were positively correlated with sesquiterpene classes but negatively with monoterpenes, suggesting tissue-specific regulation [49].

When it comes to the phenolic compounds, it was found that the accumulation of these active components in leaves of *C. coggygria* has a greater correlation with the content of inorganic elements in soil, compared to bark. In contrast to leaves, where phenolic acids and flavonoids mostly negatively correlate with inorganic elements, in the bark of *C. coggygria* greater correlation was found with total phenolics and total flavonoids. Content of catechin in leaves and gallic acid in both leaves and bark of *C. coggygria* has positive correlation with PAL activity. The leaf content of rutin and total flavonoids positively correlated with leaf area and MDA, whereas bark phenolics showed opposite trends, suggesting different allocation strategies under nutrient and stress variation [50].

The total antioxidant activity tested in extracts (TAA/e) and in essential oils (TAA/eo) of *C. coggygria* leaves was not significantly influenced by soil inorganic element content. It should be taken into account that TAA is expressed as IC_50_ value, meaning that the lower values present better antioxidant activity where negative correlation is expected for better relations among tested components. For both TAA/e and TAA/eo in leaves, the negative correlation was with leaf area, MDA, content of sesquiterpene hydrocarbons and oxygenated sesquiterpenes, and total flavonoids. Positive correlation was observed among leaf TAA/e and PAL, monoterpene hydrocarbons, and gallic acid, as well as leaf TAA/eo with monoterpene hydrocarbons, oxygenated monoterpenes, and essential oil yield. In contrast to the leaves, TAA/e and TAA/eo of *C. coggygria* bark were influenced by soil inorganic elements—positive correlation was observed among TAA/e and soil Fe, Mn, and Ni content, and among TAA/eo and soil Al, K, and S content. Bark TAA/e was negatively correlated with MDA and oxygenated sesquiterpenes, while positive correlation was shown for TAA/e and monoterpene hydrocarbons, catechin, and rutin. Bark TAA/eo was negatively correlated with PAL activity and content of oxygenated monoterpenes, gallic acid and catechin while being positively correlated with leaf area and MDA. These findings align with Zlatić et al. [45], who emphasized that inorganic elements directly affect medicinal plant quality by influencing bioactive compound biosynthesis. The authors pointed out that, although the inorganic elements are mandatory for growth and development of plants, they directly affect quality of medicinal plants through the formation and accumulation of active components, and therefore, detection and further analysis of inorganic element content in plants and soil are of great importance in ensuring the quality of medicinal plants.

## 3. Materials and Methods

### 3.1. Plant Material

**Figure 9:**
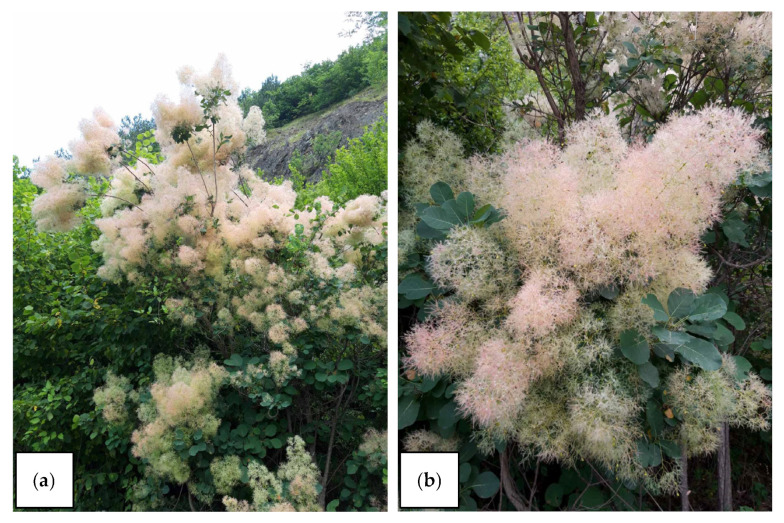
*Cotinus coggygria* during flowering period (**a**); *Cotinus coggygria* flowering twigs (**b**) (photo M. Stanković—Ibar River Gorge, Serbia).

The leaf and bark samples of *C. coggygria* were collected during the flowering period (Figure 9) from six different localities in Serbia. At each locality, twenty shoots were sampled from ten individual plants, within an area of approximately 1000 m^2^, ensuring spatial distribution and avoiding sampling from the same root system. All samples were collected from ecologically representative and visually homogeneous microsites. Plant material was taxonomically identified at the Department of Biology and Ecology, Faculty of Science, University of Kragujevac, Serbia (voucher specimen no. 139/24-1). Climatic parameters for each locality were obtained from the WorldClim database (version 2.1) and are presented in Table 7 [51]. The main ecological and geographical characteristics of the sampling sites are summarized in Table 8.

### 3.2. Reagents and Standards

All chemicals and reagents used in this study were of analytical or HPLC grade. Nitric acid (HNO_3_, 70%) and hydrogen peroxide (H_2_O_2_, 30%) were obtained from Sigma Aldrich, St. Louis, MO, USA for microwave digestion. Methanol, acetonitrile (HPLC grade), and acetic acid (99%) were purchased from Carlo Erba Reagenti (Milan, Italy), and dimethyl sulfoxide (DMSO) from Honeywell, Tokyo, Japan.

Three multi-elemental plasma standard solutions were used to prepare calibration solutions for ICP-OES measurement: Multi-Element Plasma Standard Solution 4, Specpure^®^, 1000 µg mL^−1^ (Alfa Aesar GmbH & Co KG, Karlsruhe, Germany), ILM 05.2 ICS Stock 1 and SS-Low Level Elements ICV Stock (VHG Labs, Inc.—Part of LGC Standards, Manchester, NH 03103, USA).

For HPLC analysis, 22 phenolic and related standards (gallic acid, catechin, chlorogenic acid, *p*–OH benzoic acid, vanillic acid, epicatechin, syringic acid, 3–OH benzoic acid, 3–OH–4–MeO benzaldehyde, *p*–coumaric acid, rutin, sinapinic acid, *t*–ferulic acid, naringin, 2,3–diMeO benzoic acid, benzoic acid, *o*–coumaric acid, quercetin, harpagoside, *t*–cinnamic acid, naringenin, and carvacrol) were obtained from Sigma Aldrich (Milan, Italy).

Reagents for biochemical assays included thiobarbituric acid (TBA) and 20% trichloroacetic acid (TCA) for MDA determination; 50 mM Tris-HCl buffer (pH 8.8), 10% polyvinylpolypyrrolidone (PVPP), 0.1 mM EDTA, and 20 mM L-phenylalanine for PAL enzyme activity (purchased from Merck, Darmstadt, Germany); Folin–Ciocalteu reagent (10%) and sodium bicarbonate (NaHCO_3_, 7.5%) for total phenolic content (TPC); and 2% aluminum chloride (AlCl_3_) for flavonoid content (FC) (purchased from Fluka Chemie AG, Buchs, Switzerland).

The DPPH reagent (1,1-diphenyl-2-picrylhydrazyl) used for antioxidant activity assays was obtained from Sigma Chemicals Co., St Louis, MO, USA.

### 3.3. Soil Sampling

In addition to leaves and bark samples of the *C. coggygria*, soil was sampled from the same localities for a comparative analysis of macro- and microelements. The soil sampling process followed the standard procedure [52]. Soil samples were collected at a depth of 20 cm in the root zone of each sampled individual, with three independent samples collected for each. The natural air-drying process of soil samples at room temperature took one month. After drying, the samples were pulverized, sieved through a 2 mm mesh, and stored in labeled paper bags for further analysis.

### 3.4. Content of Major and Trace Elements

#### 3.4.1. Microwave Digestion

The digestion of samples was performed on an Advanced Microwave Digestion System (ETHOS 1, Milestone, Sorisole, Italy) using an HPR-1000/10S high-pressure segmented rotor. About 0.5 g of samples was precisely weighed with accuracy ± 0.1 mg and mixed with 10 mL HNO_3_ (70 wt.%, ACS reagent, Sigma Aldrich) and 2 mL H_2_O_2_ (30 wt.%, ACS reagent, Sigma Aldrich), and then heated with microwave energy for 30 min. The temperature was gradually raised to 200 °C in the first 10 min, remained at 200 °C in the next 20 min, and then decreased rapidly to room temperature. After cooling, the solution was diluted to a fixed volume into a volumetric flask of 25 mL with ultrapure water. Ultrapure water with a resistivity of 18.2 MΩ·cm (equal to 0.05 µS/cm) was prepared using a Barnstead™ GenPure™ Pro (Thermo Scientific, Bremen, Germany).

#### 3.4.2. Determination of the Content of Elements

The contents of elements (Al, As, B, Ba, Ca, Cd, Co, Cr, Cu, Fe, K, Li, Mg, Mn, Na, Ni, P, Pb, S, Se, and Zn) were determined by inductively coupled plasma optical emission spectrometry (ICP-OES). ICP-OES measurement was performed using a Thermo Scientific iCAP 6500 Duo ICP (Thermo Fisher Scientific, Cambridge, UK). For each digested sample, the ICP-OES measurement was carried out in duplicate (*n* = 3). Quality control was performed on the analytical process using EPA Method 200.7 LPC Solution certified reference material (CRM) for 30 analytes at various contents (ULTRA Scientific, North Kongstown, Rhode Island, RI, USA), which indicated that the resulting contents were within 96–104%.

### 3.5. Variations of Leaves

A total of 180 leaves were used to determine patterns in the morphometric characteristics of *C. coggygria* leaves. Freshly sampled leaves located in the middle zone of the shoot were selected for scanning. Thirty leaves were randomly chosen from individuals within one population. Fresh leaves were scanned at a resolution of 600 dpi (Epson Perfection V19) to determine the length, width, and surface area of the leaf blade, as well as the length of the petiole.

### 3.6. Determination of Lipid Peroxidation

The thiobarbituric acid (TBA) reaction was used to extract and further evaluate the lipid peroxidation marker MDA in *C. coggygria* leaves, using the Heath and Packer [53] protocol modified by Hu et al. [54]. Leaf enzyme solution, 0.5% TBA, and 20% trichloroacetic acid were all added to the mixture. A spectrophotometric measurement of the absorbance at 532 and 600 nm was made after 30 min of incubation at 95 °C and 10 min of centrifugation at 12,000 rpm at 20 °C. The MDA content was expressed as µmol g^−1^ FW.

### 3.7. Phenylalanine Ammonium Lyase (PAL) Activity

According to He and Gao [55], the enzyme extraction was carried out from leaf tissue of *C. coggygria* using 50 mM Tris-HCl (pH 8.8) with 10% polyvinylpolypyrrolidone and 0.1 mM EDTA. The enzyme extract collected after centrifugation (20 min at 12,000 rpm) was then combined with 20 mM l-phenylalanine and 50 mM Tris-HCl buffer, and it was incubated for 30 min at 30 °C. Ten percent trichloroacetic acid was used to stop the process. PAL activity was defined as the amount of enzyme that, at 30 °C, could generate 1 nmol of trans-cinnamic acid from the substrate phenylalanine per minute [56].

### 3.8. Secondary Metabolite Extraction and Analyses

The leaves and bark of *C. coggygria* were washed with distilled water to thoroughly remove external contaminants. After air-drying naturally in a dark room at room temperature, the samples were pulverized. Dried plant material (20 g) was pulverized using a blender, and extraction was performed using the Soxhlet system. The pulverized plant material was transferred to an extraction thimble and placed in a desiccator, after which extraction with methanol was carried out. The leaf and bark extracts were evaporated in a rotary vacuum evaporator, then transferred to labeled glass vials and stored at 4 °C until further analysis.

### 3.9. Determination of Individual Phenolic Compounds

#### 3.9.1. Sample Preparation

Samples for HPLC–PDA analysis were prepared as follows: plant extracts were weighed on analytical balance and solubilized in mobile phases A (milliQ water + acetic acid)/B (acetonitrile + acetic acid) (93:7, *v*:*v*). The samples were prepared at content of 1 mg µL^−1^. All samples were vortexed for 1/2 min and sonicated for 10 min and then the supernatant was injected (20 µL) into the HPLC system for the analysis.

#### 3.9.2. HPLC Conditions

HPLC analyses were performed following a previously validated method by Locatelli et al. [57] and Ferrante et al. [58]. The instrument configuration involved a Waters liquid chromatograph equipped with a model 600 solvent pump and a 2996 photodiode array detector (PDA). Empower v.2 Software (Waters Spa, Milford, MA, USA) was used for data acquisition and elaboration. A C18 reversed-phase packing column (Prodigy ODS (3), 4.6 mm× 150 mm, 5 μm; Phenomenex, Torrance, CA, USA) was used for the separation and the column was thermostated at 30 ± 1 °C using a Jetstream 2 Plus (Waters Corporation, Milford, MA, USA). column oven. The UV/Vis acquisition wavelength was set in the range of 200–500 nm. The quantitative analyses were achieved at maximum wavelength for each compound. The injection volume was 20 μL. The mobile phase was degassed directly online by using Biotech DEGASi, mod. Compact (LabService, Anzola dell’Emilia, Bologna, Italy). Gradient elution was performed using the mobile phase water/acetonitrile (93:7, *v*:*v*, 3% acetic acid).

### 3.10. Determination of Total Phenolic Content and Flavonoid Content

According to Mihailović et al. [59], the total phenolic content (TPC) and flavonoid content (FC) were measured in methanolic extracts made from dried leaf and bark samples. For the preparation of the extracts used in the analyses, 10 g of composite plant material was used. For TPC, 10% Folin–Ciocalteu reagent and 7.5% NaHCO_3_ were used. A blank was concomitantly prepared, containing 0.5 mL methanol, 2.5 mL 10% Folin–Ciocalteu reagent dissolved in water and 2.5 mL of 7.5% of NaHCO_3_. The absorbance was measured at 765 nm after an incubation period of 15 min at 45 °C. Based on the calibration curve, the gallic acid equivalent (mg of GAE g^−1^ of extract) was used to express the TPC. The FC was measured using 2% AlCl_3_. The control sample contained all the reagents except the extract. The absorbance at 415 nm was measured using a UV–Visible spectrophotometer (Jenway 6105, Bibby Scientific Limited, Staffordshire, UK) following a one-hour incubation period at room temperature. The rutin equivalent (mg of RUE g^−1^ of extract) was used to express the FC based on the calibration curve constructed.

### 3.11. Determination of DPPH Free Radical Scavenging Capacity of Samples

The ability of DPPH (1,1-diphenyl-2-picrylhydrazyl) to scavenge radicals was used to determine the overall antioxidant activity for both extracts and essential oils [60]. The solutions of plant extracts or essential oils were prepared in methanol. Diluted solutions (1 mL of each) were mixed with 1 mL of DPPH methanolic solution. After the addition of the solution and incubation of the sample in darkness at room temperature for 30 min, the absorbance was measured at a wavelength of 517 nm. The control sample contained all the reagents except the extract. The IC_50_ values of extracts (μg mL^−1^) and essential oils (%) were estimated from the percentage inhibition versus sigmoidal curve, using a non-linear regression algorithm: % Inhibition = [(A_control − A_sample)/A_control] × 100. According to the data shown, the antioxidant efficacy increases as IC_50_ values decrease.

### 3.12. Chemical Analysis of Essential Oil

Essential oils were isolated via hydrodistillation using a Clevenger-type apparatus, following the protocol outlined in the European Pharmacopoeia 11.0 [61]. For each sample, 100 g of fresh leaves or bark was combined with 1 L of distilled water and subjected to hydrodistillation for 3 h. The resulting oil was dried over anhydrous sodium sulfate and stored in sterile, dark glass vials at 4 °C. Essential oil yield was determined volumetrically and expressed as milliliters of oil per 100 g of plant material (v/m).

The chemical composition of the essential oils was analyzed using gas chromatography–mass spectrometry (GC/MS). Analyses were carried out on a Shimadzu GCMS-QP2010 Ultra system using GCMSsolution software (Version 2.x; Shimadzu Corporation, Kyoto, Japan), equipped with a flame ionization detector (FID) and coupled to a GC-2010 gas chromatograph. An InertCap 5 capillary column (60.0 m × 0.25 mm i.d. × 0.25 µm film thickness) was used for compound separation. Helium was employed as the carrier gas at a linear velocity of 35.2 cm/s and a split ratio of 1:5. The GC oven temperature was initially set at 60 °C (held for 4 min), then programmed to increase at a rate of 4 °C/min to 280 °C, where it was held for an additional 10 min. Injector and detector temperatures were set at 250 °C and 300 °C, respectively, while the ion source temperature was maintained at 200 °C.

Compound identification was carried out by comparing the obtained mass spectra and calculated retention indices (RI) with those of authentic standards and data available in the NIST and Wiley mass spectral libraries. Retention indices were determined using the Kovats Index (KI) method, relative to a homologous series of C8–C40 n-alkanes analyzed under identical chromatographic conditions on the InertCap 5 column. Identification was further supported by automated library searches (PBM, NIST, AMDIS) and comparison with values reported in the literature [62].

### 3.13. Statistical Analysis

All experimental measurements were carried out in triplicate and are expressed as the average of three analyses ± SD. Results were analyzed using IBM SPSS Statistics, ver. 19 (Armonk, NY, USA: IBM Corp). Analysis of variance (ANOVA) and Tukey’s test were performed with a level of confidence of 95%. PCA was performed using XLStat (version 2024.2, Addinsoft, New York, NY, USA) software. A Pearson correlation matrix and data n-standardization were used to perform PCA according to Cañas et al. [63].

## 4. Conclusions

This multidisciplinary study highlights significant eco-physiological, morphological, and chemical variability among natural populations of *Cotinus coggygria* across diverse habitats. The accumulation of macro- and microelements in leaves and bark showed a clear relationship with soil composition, influencing key physiological and biochemical processes. Notably, variations in oxidative stress markers (MDA), PAL activity, and leaf morphology reflect the species adaptive responses to differing environmental conditions. Quantitative analysis revealed considerable differences in phenolic content, particularly catechin concentration, as well as antioxidant potential across populations. Observed differences in essential oil composition may reflect the influence of environmental conditions specific to each geographic location, with monoterpene hydrocarbons predominating and limonene and α-pinene as key constituents. Principal component and correlation analyses confirmed a strong link between habitat-specific abiotic factors and the chemical profile of the plant. Overall, the findings underscore the ecological plasticity of *C. coggygria* and provide a valuable basis for further studies. Future research should focus on genetic diversity and detailed pharmacological investigations to better understand the bioactive potential of the species. Additionally, exploring sustainable strategies for cultivation and extraction could support future conservation and utilization efforts.

## Figures and Tables

**Table 1:** The quantity of major and trace elements in soil samples from different localities.

Element	Sample	BG *	IG	OKG	GRG	DG	DSD
Al	Soil	8890.15 ± 36.0 b	5284.07 ± 4.3 c	12,062.67 ± 18.0 a	12,650.60 ± 13.0 a	328.32 ± 20.0 d	5369.29 ± 8.0 c
	Leaves	7.10 ± 1.8 bc	17.30 ± 2.6 a	5.26 ± 0.2 c	9.76 ± 1.4 b	11.79 ± 2.8 ab	11.79 ± 2.9 ab
	Bark	17.39 ± 1.9 b	20.89 ± 0.8 b	8.52 ± 1.20 c	29.35 ± 2.9 a	14.91 ± 2.2 b	15.04 ± 3.1 b
As	Soil	1.48 ± 1.15 c	1.93 ± 0.19 c	19.37 ± 1.0 a	5.04 ± 0.20 b	18.42 ± 1.7 a	3.73 ± 0.06 c
	Leaves	n.d. **	n.d.	n.d.	n.d.	n.d.	n.d.
	Bark	n.d.	n.d.	n.d.	n.d.	n.d.	n.d.
B	Soil	6.46 ± 0.40 a	6.88 ± 1.6 a	2.44 ± 0.46 bc	3.94 ± 0.62 b	0.88 ± 0.08 cd	1.46 ± 0.12 c
	Leaves	13.01 ± 2.4 c	16.40 ± 0.3 b	13.50 ± 0.3 c	14.68 ± 2.0 c	20.88 ± 0.8 a	12.40 ± 0.9 c
	Bark	12.75 ± 1.2 b	13.44 ± 1.6 b	13.74 ± 0.1 b	13.90 ± 1.3 b	15.29 ± 0.8 ab	16.69 ± 0.5 a
Ba	Soil	22.35 ± 10.6 c	42.83 ± 5.3 b	53.13 ± 3.0 a	45.65 ± 2.90 ab	2.18 ± 0.12 d	21.82 ± 3.1 c
	Leaves	1.33 ± 0.39 e	6.65 ± 1.1 d	23.88 ± 3.5 a	12.99 ± 2.7 c	16.05 ± 1.8 b	7.61 ± 1.3 cd
	Bark	9.66 ± 0.5 c	39.63 ± 2.1 a	43.25 ± 3.6 a	25.32 ± 3.3 b	27.01 ± 1.7 b	30.74 ± 2.5 b
Ca	Soil	6912.99 ± 291.0 e	4553.54 ± 182.0 f	64,405.99 ± 581.0 c	151,449.55 ± 90.0 b	365,118.77 ± 1081.0 a	11,831.88 ± 483.0 d
	Leaves	5022.17 ± 78.0 d	4369.37 ± 69.0 d	13,165.68 ± 80.0 b	12,116.14 ± 74.0 bc	16,562.80 ± 81.0 a	9911.79 ± 69.0 c
	Bark	6092.84 ± 174.0 d	5624.63 ± 127.0 d	14,163.57 ± 147.0 a	13,038.63 ± 136.0 b	13,945.09 ± 125.0 a	12,301.82 ± 122.0 bc
Cd	Soil	4.28 ± 0.41 a	4.29 ± 0.19 a	2.05 ± 0.07 b	1.35 ± 0.11 bc	0.37 ± 0.06 d	1.09 ± 0.05 c
	Leaves	n.d.	0.02 ± 0.01 a	0.01 ± 0.01 a	0.01 ± 0.01 a	0.01 ± 0.01 a	0.010 ± 0.01 a
	Bark	0.03 ± 0.01 b	0.03 ± 0.01 b	0.02 ± 0.01 b	0.05 ± 0.01 a	0.03 ± 0.01 b	0.04 ± 0.01 ab
Co	Soil	96.36 ± 10.0 a	63.96 ± 3.0 b	10.30 ± 0.5 c	5.53 ± 0.1 c	2.36 ± 0.28 c	5.55 ± 0.6 c
	Leaves	n.d.	n.d.	n.d.	n.d.	n.d.	n.d.
	Bark	0.17 ± 0.03 a	0.15 ± 0.02 a	n.d.	n.d.	0.02 ± 0.01 b	n.d.
Cr	Soil	551.15 ± 53.0 a	567.64 ± 131.0 a	37.02 ± 2.7 b	24.83 ± 2.1 b	6.59 ± 0.20 c	20.04 ± 3.4 b
	Leaves	0.36 ± 0.05 b	2.61 ± 0.08 a	0.92 ± 0.18 c	n.d.	0.05 ± 0.01 d	0.41 ± 0.04 b
	Bark	0.75 ± 0.10 c	1.12 ± 0.22 b	0.63 ± 0.08 c	0.79 ± 0.09 c	1.45 ± 0.02 a	0.53 ± 0.04 c
Cu	Soil	30.14 ± 2.40 b	40.67 ± 0.70 a	26.01 ± 1.2 c	25.57 ± 1.5 c	4.86 ± 0.38 e	13.69 ± 0.1 d
	Leaves	2.11 ± 0.20 c	3.26 ± 0.03 b	2.42 ± 0.19 c	7.19 ± 0.2 a	2.85 ± 0.19 b	2.77 ± 0.33 bc
	Bark	3.02 ± 0.66 b	3.35 ± 0.31 b	2.65 ± 0.43 b	6.43 ± 0.5 a	3.12 ± 0.12 b	3.71 ± 0.82 b
Fe	Soil	60,683.28 ± 104.0 a	55,621.77 ± 104.0 b	23,423.76 ± 178.0 c	15,352.03 ± 60.0 d	1807.83 ± 18.0 f	10,937.80 ± 15.0 e
	Leaves	30.20 ± 2.4 c	56.26 ± 11.0 a	22.82 ± 2.3 c	45.88 ± 5.3 b	38.00 ± 2.8 b	40.30 ± 0.1 b
	Bark	47.27 ± 8.9 b	70.60 ± 3.0 a	21.31 ± 2.4 c	62.65 ± 13.0 a	32.99 ± 4.6 b	40.85 ± 4.9 b
K	Soil	1041.90 ± 21.0 bc	553.73 ± 6.0 d	1077.17 ± 1.0 b	2083.49 ± 10.0 a	184.04 ± 6.40 e	609.81 ± 11.0 d
	Leaves	1987.18 ± 160.0 d	2373.01 ± 210.0 cd	3143.0 ± 186.0 b	2489.76 ± 166.0 c	3484.91 ± 153.0 a	2918.76 ± 199.0 bc
	Bark	2867.99 ± 84.0 c	3112.29 ± 116.0 bc	2765.29 ± 178.0 c	3592.78 ± 114.0 b	4647.88 ± 150.0 a	3659.33 ± 108.0 b
Li	Soil	14.54 ± 0.68 e	9.62 ± 0.17 f	78.11 ± 0.3 c	131.21 ± 0.7 b	221.86 ± 3.70 a	21.03 ± 0.37 d
	Leaves	3.24 ± 0.56 cd	2.88 ± 0.26 d	6.30 ± 0.5 ab	5.68 ± 0.6 b	8.05 ± 1.2 a	4.65 ± 0.8 bc
	Bark	3.79 ± 1.01 c	3.56 ± 0.40 c	6.44 ± 0.6 a	5.69 ± 0.5 ab	6.45 ± 0.1 a	5.42 ± 0.2 b
Mg	Soil	1428.84 ± 149.0 a	1484.41 ± 117.0 a	856.88 ± 124.0 c	900.32 ± 93.0 bc	661.99 ± 60.0 d	1119.05 ± 60.0 b
	Leaves	790.92 ± 66.0 a	1008.24 ± 136.0 a	835.31 ±87.0 a	793.32 ± 74.0 a	917.23 ± 72.0 a	825.44 ± 82.0 a
	Bark	570.12 ± 85.0 bc	897.47 ± 62.0 a	508.11 ± 78.3 c	626.94 ± 91.0 b	630.54 ± 96.0 b	662.90 ± 95.0 b
Mn	Soil	887.60 ± 8.1 a	893.92 ± 8.2 a	446.77 ± 10.2 b	289.63 ± 3.0 c	215.59 ± 0.80 c	258.65 ± 4.2 c
	Leaves	6.80 ± 1.4 cd	4.61 ± 1.34 d	8.09 ± 0.6 c	30.58 ± 2.8 a	15.26 ± 2.1 b	11.84 ± 1.2 b
	Bark	7.10 ± 0.1 cd	6.51 ± 0.85 d	4.54 ± 0.80 e	8.77 ± 1.2 bc	10.14 ± 2.2 b	12.90 ± 1.0 a
Na	Soil	108.58 ± 23.0 a	112.48 ± 49.0 a	n.d.	n.d.	n.d.	41.53 ± 8.80 b
	Leaves	8.74 ± 1.9 c	13.11 ± 2.0 b	10.80 ± 1.9 bc	22.66 ± 2.6 a	8.91 ± 1.1 c	7.75 ± 1.6 d
	Bark	88.35 ± 9.0 b	71.15 ± 7.0 b	24.03 ± 4.6 c	187.69 ± 16.0 a	20.78 ± 3.2 c	28.36 ± 4.1 c
Ni	Soil	850.32 ± 99.0 a	887.96 ± 2.0 a	75.58 ± 4.0 b	15.08 ± 0.1 c	5.15 ± 0.4 d	19.73 ± 0.1 c
	Leaves	1.58 ± 0.61 b	2.18 ± 0.15 a	0.26 ± 0.04 c	0.26 ± 0.03 c	0.29 ± 0.03 c	0.20 ± 0.04 c
	Bark	2.18 ± 0.35 a	2.13 ± 0.59 a	0.76 ± 0.07 b	0.58 ± 0.06 b	0.45 ± 0.09 bc	0.37 ± 0.02 c
P	Soil	323.28 ± 37.0 d	1988.97 ± 38.0 a	915.34 ± 25.0 b	522.87 ± 27.0 c	458.28 ± 3.0 cd	864.43 ± 60.0 b
	Leaves	1712.45 ± 49.0 c	1897.64 ± 53.0 bc	1738.66 ± 45.0 c	1530.34 ± 33.0 d	2407.98 ± 69.0 a	1508.36 ± 48.0 d
	Bark	1250.48 ± 85.0 a	1298.60 ± 53.0 a	856.46 ± 51.0 b	1346.08 ± 45.0 a	1486.51 ± 42.0 a	1353.05 ± 21.0 a
Pb	Soil	14.67 ± 3.2 b	63.02 ± 3.0 a	17.37 ± 2.0 b	8.33 ± 0.1 c	1.74 ± 0.21 d	8.29 ± 0.3 c
	Leaves	0.05 ± 0.01 a	0.06 ± 0.01 a	n.d.	0.10 ± 0.02 a	0.01 ± 0.01 a	0.01 ± 0.01 a
	Bark	0.64 ± 0.12 b	0.61 ± 0.12 b	0.41 ± 0.06 c	0.90 ± 0.04 a	0.25 ± 0.01 cd	0.05 ± 0.01 d
S	Soil	247.36 ± 25.0 c	703.71 ± 31.0 a	386.86 ± 34.0 b	394.95 ± 23.0 b	183.95 ± 24.0 c	231.05 ± 5.0 c
	Leaves	961.54 ± 10.0 a	1052.81 ± 9.0 a	1095.66 ± 19.0 a	921.27 ± 11.0 a	1067.67 ± 10.0 a	972.61 ± 18.0 a
	Bark	448.31 ± 64.0 a	423.28 ± 11.0 a	363.54 ± 4.0 b	403.68 ± 6.0 a	406.36 ± 17.0 a	433.96 ± 26.0 a
Se	Soil	n.d. *	n.d.	n.d.	n.d.	n.d.	n.d.
	Leaves	0.46 ± 0.1 a	0.46 ± 0.11 a	0.42 ± 0.10 a	0.51 ± 0.09 a	0.40 ± 0.08 a	0.52 ± 0.10 a
	Bark	0.51 ± 0.04 b	0.73 ± 0.03 a	0.80 ± 0.11 a	0.60 ± 0.02 b	0.82 ± 0.10 a	0.62 ± 0.10 b
Zn	Soil	89.89 ± 9.0 a	86.96 ± 1.0 a	61.46 ± 3.0 b	47.49 ± 2.0 c	27.22 ± 0.4 d	35.14 ± 0.1 cd
	Leaves	13.49 ± 0.8 b	15.40 ± 0.1 a	12.72 ± 0.7 b	16.20 ± 0.2 a	10.07 ± 0.1 bc	9.59 ± 0.4 c
	Bark	31.95 ± 4.5 b	39.05 ± 1.1 a	21.70 ± 0.3 c	28.58 ± 1.8 b	17.79 ± 1.4 c	34.15 ± 0.2 ab

* Serpentinite habitat (BG, IG); limestone habitat (OKG, GRG, DG); sandy habitat (DSD). ** n.d. <0.005 mg/kg. Means sharing the same letter in a column do not differ significantly by Tukey’s test (*p* ≥ 0.05).

**Table 2:** Morphological characteristics of *C. coggygria* leaves from examined populations.

Sample	Leaf Blade Length (mm)	Leaf Plate Width (mm)	The Surface of the Leaf Plate (mm^2^)	Petiole Length (mm)
BG	71.21 ± 6.27 b	46.24 ± 4.32 c	2075.99 ± 382.27 c	32.47 ± 11.89 a
IG	67.56 ± 7.57 cd	45.18 ± 7.56 c	2479.35 ± 534.35 b	23.43 ± 6.86 b
OKG	65.54 ± 9.69 d	45.45 ± 5.79 c	2453.22 ± 578.44 b	15.95 ± 5.83 c
GRG	76.71 ± 8.00 a	57.76 ± 6.78 a	3550.65 ± 653.07 a	23.29 ± 5.59 b
DG	64.37 ± 5.59 d	48.93 ± 5.10 b	2552.70 ± 452.22 b	20.99 ± 8.01 b
DSD	70.89 ± 8.15 bc	46.22 ± 5.88 bc	2374.44 ± 509.65 b	22.52 ± 8.72 b

Means sharing the same letter in a column do not differ significantly by Tukey’s test (*p* ≥ 0.05).

**Table 3:** Total amounts (mg g^−1^) of phenolics in leaves and bark of *C. coggygria*.

		BG *	IG	OKG	GRG	DG	DSD
Leaves							
	Gallic acid	26.99 ± 1.35 a	6.77 ± 0.41 d	22.60 ±1.13 b	18.70 ±1.87 c	17.01 ± 1.02 c	26.20 ± 1.57 a
	Catechin	390.29 ± 23.41 f	444.00 ± 22.20 d	1699.44 ± 84.97 c	420.21 ± 25.21 e	2268.73 ± 113.41 a	2200.10 ± 110.01 b
	Chlorogenic acid	13.18 ± 0.79 a	BLQ **	9.17 ±0.46 b	/	6.01 ± 0.36 c	2.33 ± 0.14 d
	Epicatechin	19.69 ± 1.18 a	/	11.04 ± 0.66 b	5.99 ± 0.30 c	5.83 ± 0.29 c	/
	Syringic acid	/	/	/	/	/	/
	Rutin	60.49 ± 3.63 cd	37.12 ± 2.23 e	56.88 ± 3.41 d	145.25 ± 8.72 a	72.73 ± 4.37 bc	84.46 ± 5.07 b
	*o*-coumaric acid	49.03 ± 2.45 a	27.72 ± 1.66 bc	24.62 ± 1.48 cd	30.58 ± 1.83 b	22.39 ± 1.34 d	50.98 ± 3.06 a
	Total	559.67 ± 27.98 d	515.86 ± 30.95 d	1823.75 ± 109.43 b	620.74 ± 31.04 c	2392.69 ± 119.63 a	2364.07 ± 118.20 a
Bark							
	Gallic acid	13.18 ± 0.79 a	7.07 ± 0.42 b	6.22 ± 0.44 b	4.77 ± 0.29 b	11.41 ± 1.03 a	12.84 ± 1.03 a
	Catechin	308.69 ± 15.43 a	164.64 ± 9.88 c	183.56 ± 11.01 b	113.26 ± 6.80 e	299.63 ± 14.98 a	145.71 ± 11.66 d
	Chlorogenic acid	/	/	/	/	/	/
	Epicatechin	/	64.98 ± 3.90 b	113.91 ± 6.83 a	/	114.51 ± 6.87 a	108.10 ± 6.49 a
	Syringic acid	58.58 (±3.51) a	/	/	34.65 (±2.08) b	/	/
	Rutin	8.91 ± 0.53 a	2.90 ± 0.17 b	7.20 ± 0.43 a	3.43 ± 0.21 b	3.94 ± 0.24 b	1.63 ± 0.10 b
	*o*-coumaric acid	/	/	/	/	/	/
	Total	389.36 ± 19.47 b	239.58 ± 14.37 e	310.89 ± 18.65 c	156.10 ± 9.37 f	429.50 ± 21.48 a	268.29 ± 13.41 d

* Serpentinite habitat (BG, IG); limestone habitat (OKG, GRG, DG); sandy habitat (DSD). ** BLQ (Below the Limit of Quantification). Means sharing the same letter in a column do not differ significantly by Tukey’s test (*p* ≥ 0.05).

**Table 4:** Total amount of phenolic compounds (TPC), total amount of flavonoids (TFC), total antioxidant activity of extracts (TAA/e), and total antioxidant activity of essential oils (TAA/eo) from leaves and bark of *C. coggygria*.

Sample		TPC (mg GAE g^−1^ of Sample)	TFC (mg RUE g^−1^ of Sample)	TAA/e (µg mL^−1^)	TAA/eo (%)
BG *	Leaves	472.96 ± 3.42 b	57.84 ± 0.33 ab	1219.76 ± 6.52 d	3.89 ± 0.40 a
IG		526.47 ± 5.12 a	60.56 ± 0.00 ab	548.50 ± 5.14 b	2.69 ± 0.25 b
OKG		454.45 ± 2.85 b	54.99 ± 0.27 b	622.59 ± 4.82 b	3.84 ± 0.13 a
GRG		452.56 ± 4.35 b	72.43 ± 0.40 a	210.56 ± 2.16 a	0.70 ± 0.06 c
DG		473.05 ± 6.69 b	64.42 ± 0.20 ab	801.24 ± 5.81 c	2.72 ± 0.28 b
DSD		510.53 ± 6.80 a	63.90 ± 0.36 ab	897.11 ± 6.32 c	1.43 ± 0.15 c
BG	Bark	218.99 ± 4.09 a	41.30 ± 0.49 a	143.07 ± 5.62 d	4.42 ± 0.56 d
IG		195.07 ± 3.44 b	37.33 ± 0.13 ab	105.89 ± 3.18 c	5.61 ± 0.21 abc
OKG		187.86 ± 1.88 b	22.85 ± 0.10 c	106.78 ± 4.56 c	5.90 ± 0.45 ab
GRG		203.26 ± 4.71 a	37.39 ± 0.27 ab	86.24 ± 3.62 b	6.31 ± 0.63 a
DG		186.32 ± 7.43 b	26.91 ± 0.16 c	116.35 ± 2.85 c	4.83 ± 0.23 cd
DSD		190.82 ± 1.03 b	33.42 ± 0.08 b	31.48 ± 1.52 a	4.98 ± 0.41 bcd

* Serpentinite habitat (BG, IG); limestone habitat (OKG, GRG, DG); sandy habitat (DSD). BHT 5.75% neutralization ability of DPPH. TAA/e expressed as μg mL^−1^; TAA/eo expressed as %. Means sharing the same letter in a column do not differ significantly by Tukey’s test (*p* ≥ 0.05).

**Table 5:** Composition of the *C. coggygria* leaf essential oils (%).

	EO Constituents	KI *	RI **	BG ***	IG	OKG	GRG	DG	DSD
1	(*E*)-2-Hexanal	846	853.1	0.07	0.10	/	0.06	/	/
2	(*Z*)-3-Hexanol	850	856.9	0.15	0.17	/	0.03	/	0.07
3	*α*-Pinene	932	938.6	1.81	2.09	2.77	8.62	3.93	6.33
4	Camphene	946	954.4	0.10	/	/	0.14	0.14	0.20
5	*β*-Pinene	974	982.8	0.36	0.19	0.37	1.83	0.65	0.89
6	Myrcene	988	991.9	1.23	1.16	2.33	10.98	1.22	9.46
7	(*E*)-3-Hexenyl acetate	1001	1005.9	0.50	0.37	0.30	0.35	1.64	0.40
8	*α*-Phellandrene	1002	1009.0	0.07	/	0.07	0.07	0.08	0.07
9	*δ*-3-Carene	1008	1015.3	0.20	0.18	0.30	0.13	0.26	0.11
10	*α*-Terpinene	1014	1021.2	0.07	/	0.09	0.31	0.08	/
11	Limonene	1024	1036.5	59.17	61.86	68.73	31.74	56.01	37.15
12	*cis*-*β*-Ocimene	1032	1040.3	22.30	20.28	10.16	27.98	19.41	31.85
13	*trans*-*β*-Ocimene	1044	1050.0	5.22	5.05	2.93	6.19	4.70	6.53
14	Terpinolene	1086	1093.8	5.56	4.74	8.51	3.86	6.54	2.43
15	Linalool	1095	1101.0	0.10	0.09	0.09	0.02	0.14	0.12
16	*n*-Nonanal	1100	1103.9	0.10	0.09	0.14	0.08	0.16	0.07
17	*allo*-Ocimene	1128	1130.6	0.32	0.31	0.16	0.45	0.27	0.46
18	(*Z*)-3-Hexenyl butanoate	1184	1185.0	0.08	/	/	0.09	0.08	0.08
19	*p*-Cymen-8-ol	1179	1190.8	0.21	0.20	0.36	0.13	0.18	0.07
20	*α*-Terpineol	1186	1197.6	0.15	0.14	0.14	0.14	0.13	0.15
21	(*Z*)-3-Hexenyl 2-methyl butanoate	1229	1232.0	/	/	/	0.05	0.07	/
22	Isobornyl acetate	1283	1293.3	0.10	0.12	0.21	0.10	0.11	/
23	*α*-Terpinyl acetate	1346	1355.7	0.24	0.26	0.47	0.15	0.36	0.15
24	*α*-Copaene	1374	1390.4	/	/	/	0.17	0.07	0.07
25	*trans*-*β*-Caryophyllene	1417	1439.5	0.67	1.74	0.77	1.96	2.73	0.92
26	*allo*-Aromadendrene	1458	1459.0	0.10	0.10	0.12	0.08	/	0.07
27	*α*-Humulene	1452	1474.0	0.08	0.12	0.08	0.25	0.19	0.15
28	*trans*-9-epi-Caryophyllene	1464	1481.8	/	/	/	0.10	/	0.08
29	*γ*-Muurolene	1478	1491.8	0.13	0.07	0.08	0.21	0.08	0.17
30	Germacrene-D	1484	1500.4	0.09	0.09	0.09	2.25	0.23	1.01
31	*γ*-Amorphene	1483	1495.9	/	/	/	0.06	/	/
32	*α*-Muurolene	1500	1514.9	0.17	0.12	0.14	0.30	/	0.23
33	*γ*-Cadinene	1513	1532.0	0.09	/	/	0.10	/	0.11
34	*δ*-Cadinene	1522	1537.9	0.23	0.10	0.14	0.39	0.16	0.36
35	Spathulenol	1577	1601.1	/	/	/	0.15	/	0.07
36	Cariophyllene oxide	1582	1609.0	0.07	0.15	0.07	0.17	0.10	0.09
	Monoterpene hydrocarbons (MH)			96.9	96.2	96.7	92.6	94.9	95.9
	Oxygenated monoterpenes (OM)			1.2	1.2	1.4	0.9	1.2	0.7
	Sesquiterpene hydrocarbons (SH)			1.6	2.3	1.4	5.9	3.5	3.2
	Oxygenated sesquiterpenes (OS)			0.1	0.2	0.1	0.3	0.1	0.2
	Total identified			99.75	99.88	99.63	99.69	99.73	99.90
	Yields (V/m)			0.25	0.25	0.20	0.175	0.20	0.15

* KI—Kovats index; ** RI—retention index relative to C8-C40 n-alkanes on InertCap5 column; *** serpentinite habitat (BG, IG); limestone habitat (OKG, GRG, DG); sandy habitat (DSD).

**Table 6:** Composition of the *C. coggygria* bark essential oils (%).

	Components	KI *	RI **	BG ***	IG	OKG	GRG	DG	DSD
1	Tricyclene	921	927.2	0.25	0.10	0.06	/	0.14	0.16
2	*α*-Thujene	924	930.5	0.42	0.10	9.70	0.86	1.14	0.97
3	*α*-Pinene	932	940.5	51.71	54.52	43.23	47.29	48.76	45.09
4	Camphene	946	954.6	1.80	0.80	0.70	0.50	1.19	1.33
5	Sabinene	969	978.1	0.40	0.45	1.73	0.51	0.54	0.47
6	*β*-Pinene	974	983.8	16.00	16.48	13.07	12.37	14.52	13.03
7	Myrcene	988	991.8	1.15	1.03	8.66	9.59	1.35	2.10
8	*α*-Phellandrene	1002	1008.8	0.18	0.13	0.52	0.25	0.38	0.68
9	*δ*-3-Carene	1008	1015.1	0.06	/	0.09	0.08	/	0.07
10	*α*-Terpinene	1014	1020.9	/	/	0.22	0.07	0.09	0.10
11	*p*-Cymene	1020	1028.7	0.32	0.23	0.24	0.25	0.06	0.14
12	Limonene	1024	1034.8	12.00	6.55	8.57	3.40	13.51	7.62
13	*β*-Phellandrene	1025	1035.7	/	3.09	3.51	3.79	/	4.38
14	*cis*-*β*-Ocimene	1032	1038.2	1.91	1.40	0.97	0.46	2.60	2.85
15	*trans*-*β*-Ocimene	1044	1048.9	0.36	0.28	0.36	0.11	0.70	0.66
16	*γ*-Terpinene	1054	1062.6	/	/	0.79	0.07	0.19	0.16
17	Terpinolene	1086	1092.9	0.75	0.26	0.97	1.41	1.07	1.51
18	Linalool	1095	1100.9	/	/	0.08	/	/	/
19	*α*-Pinene oxide	1099	1105.7	0.04	0.08	/	/	/	/
20	*allo*-Ocimene	1128	1130.6	/	/	/	/	0.06	0.08
21	*trans*-Pinocarveol	1135	1148.5	0.10	0.07	/	/	/	/
22	Terpinne-4-ol	1174	1185.2	0.20	0.10	0.40	0.08	0.14	0.22
23	*p*-Cymen-8-ol	1179	1190.9	0.08	0.15	/	0.07	/	/
24	Cryptone	1183	1194.1	0.14	/	/	0.09	/	/
25	*α*-Terpineol	1186	1197.7	0.38	0.14	0.14	0.10	0.15	0.31
26	Myrtenal	1195	1204.9	0.11	0.10	/	/	/	0.06
27	Linalool acetate	1254	1255.7	/	/	0.14	0.07	0.08	0.11
28	Bornyl acetate	1287	1293.4	0.36	0.09	/	0.07	0.59	0.65
29	Pinocarvyl acetate	1298	1309.5	0.07	/	/	/	/	/
30	*cis*-Pinocarvyl acetate	1311	1319.2	0.08	/	/	0.08	/	/
31	Terpinyl acetate	1346	1356.0	0.62	0.09	0.12	0.12	0.13	0.23
32	*α*-Copaene	1374	1390.8	0.32	0.35	0.13	0.48	0.17	0.15
33	*β*-Longipinene	1400	1404.0	0.14	0.17	/	0.24	0.10	0.22
34	*α*-Gurjunene	1409	1427.6	/	0.11	/	0.08	/	0.15
35	*trans*-*β*-Caryophyllene	1417	1439.4	0.46	0.58	0.31	0.42	0.75	0.42
36	*α*-neo-Clovene	1452	1461.3	0.56	0.09	1.24	/	/	/
37	*α*-Humulene	1452	1474.2	0.33	0.41	0.14	0.46	0.32	0.37
38	*allo*-Aromadendrene	1458	1481.8	0.10	0.20	/	0.18	0.06	0.22
39	Cadinene	1513	1492.6	0.60	0.60	0.20	0.48	0.58	0.54
40	Germacrene D	1484	1501.5	4.69	6.95	3.00	12.64	8.62	9.58
41	*β*-Selinene	1489	1507.8	0.07	0.10	/	/	/	0.11
42	*γ*-Amorphene	1495	1511.0	0.13	0.13	/	/	0.07	0.14
43	*α*-Muurolene	1500	1515.1	0.44	0.54	0.17	0.47	0.24	0.66
44	*δ*-Cadinene	1522	1538.3	0.92	1.06	0.27	0.79	0.79	1.22
45	Spathulenol	1577	1601.1	0.20	0.15	0.08	0.15	0.06	0.14
46	Viridiflorol	1592	1609.1	0.11	0.10	/	0.09	/	0.09
47	Ledol	1602	1618.0	0.10	/	0.09	/	/	0.11
48	*β*-Oplopenone	1607	1632	/	/	/	/	0.12	0.16
49	Cubenol	1645	1650.2	/	/	/	0.11	/	0.06
50	*τ*-Cadinol	1638	1661.7	0.31	0.46	/	0.39	0.11	0.55
51	*α*-Muurolol	1644	1666.1	/	/	/	/	/	0.15
52	*α*-Cadinol	1652	1676.5	0.29	0.54	/	0.55	0.18	1.10
53	Caryophylla-4(12),8(13)-dien5-*β*-ol	1639	1701.7	0.10	0.17	/	0.17	/	/
54	Amorpha-4,9-dien-2-ol	1700	1711.2	0.08	0.07	/	0.08	0.06	0.08
55	ethylhexyl -2-Salicylat	1807	1819.9	0.07	0.07	/	/	/	/
56	Longifolol acetate	1819	1893.3	0.07	0.09	/	/	/	/
	Monoterpene hydrocarbons (MH)			87.32	85.44	93.40	81.02	86.31	81.41
	Oxygenated monoterpenes (OM)			2.18	0.82	0.88	0.68	1.09	1.60
	Sesquiterpene hydrocarbons (SH)			8.75	1 1.29	5.46	16.23	11.71	13.79
	Oxygenated sesquiterpenes (OS)			1.34	1.65	0.17	1.54	0.53	2.44
	Total identified			99.59	99.21	99.92	99.47	99.64	99.24
	Yields (V/m)			0.75	0.60	1.00	0.80	0.60	0.55

* KI—Kovats index; ** RI—retention index relative to C8–C40 n-alkanes on InertCap5 column. *** Serpentinite habitat (BG, IG); limestone habitat (OKG, GRG, DG); sandy habitat (DSD).

**Table 7:** Bioclimatic parameters of the six *Cotinus coggygria* sampling localities based on WorldClim v2.1 data.

Climatic Conditions	BG *	IG	OKG	GRG	DG	DSD
Annual mean temperature (bio1)	10.6	10.0	9.8	9.8	10.0	11.5
Mean monthly temperature range (bio2)	9.9	9.8	9.5	9.8	9.3	9.7
Isothermality (bio2/bio7) (×100) (bio3)	32.7	32.5	32.1	32.5	30.9	31.9
Temperature seasonality (STD × 100) (bio4)	749.2	749.3	737.9	758.3	782.1	778.8
Max temperature of warmest month (bio5)	26.3	25.8	25.0	25.9	26.4	27.9
Min temperature of coldest month (bio6)	−3.9	−4.4	−4.5	−4.2	−3.7	−2.5
Temperature annual range (bio5–bio6) (bio7)	30.2	30.2	29.5	30.1	30.1	30.4
Mean temperature of wettest quarter (bio8)	17.8	17.1	16.8	17.2	17.5	19.0
Mean temperature of driest quarter (bio9)	2.6	1.9	1.9	1.8	1.6	3.1
Mean temperature of warmest quarter (bio10)	19.5	18.9	18.6	18.8	19.2	20.6
Mean temperature of coldest quarter (bio11)	1.0	0.4	0.4	0.2	0.0	1.4
Annual precipitation (bio12)	793	829	851	693	697	639
Precipitation of wettest month (bio13)	89	94	93	83	94	84
Precipitation of driest month (bio14)	50	52	54	44	43	41
Precipitation seasonality (CV) (bio15)	19.7	18.0	18.4	22.7	27.8	25.8
Precipitation of wettest quarter (bio16)	256	259	269	231	247	221
Precipitation of driest quarter (bio17)	158	167	170	136	132	124
Precipitation of warmest quarter (bio18)	229	230	242	205	226	201
Precipitation of coldest quarter (bio19)	172	177	185	147	144	138

* Serpentinite habitat (BG, IG); limestone habitat (OKG, GRG, DG); sandy habitat (DSD). Isothermality (bio3) is calculated as the ratio of mean diurnal temperature range (bio2) to annual temperature range (bio7) (bio2/bio7 × 100), multiplied by 100. Temperature seasonality (bio4) is calculated as the standard deviation of monthly mean temperatures (°C) multiplied by 100 (STD × 100).

**Table 8:** Overview of environmental and geographical characteristics of the sampling localities of *Cotinus coggygria* populations in Serbia, including coordinates, altitude, climate type, soil type, and dominant vegetation.

Locality/Abbreviation	Type of Habitat	Latitude and Longitude	Altitude
Brdjani Gorge/BG	Serpentinite rocky habitat	N 43.988121° E 20.417711°	305 m
Ibar Gorge/IG	Edge of pine forest, serpentinite substrate	N 43.607878° E 20.553707°	277 m
Ovčarsko-Kablarska Gorge/OKG	Edge of oak forest, limestone substrate	N 43.899992° E 20.190301°	286 m
Grza Gorge/GRG	Thermophilous rocky habitat, limestone substrate	N 43.858200° E 21.614274°	357 m
Djerdap Gorge/DG	Thermophilous rocky habitat, limestone substrate	N 44.841213° E 21.259362°	90 m
Deliblato Sands/DSD	Thermophilous sandy habitat, silicate/carbonate substrate	N 44.659712° E 21.671853°	81 m

## Data Availability

The data underlying this article will be shared upon reasonable request to the corresponding author.
